# Structural Characterization of *Dendrobium officinale* Polysaccharides and Their Regulation Effect on Intestinal Microbiota During In Vitro Fermentation

**DOI:** 10.3390/polym17060727

**Published:** 2025-03-10

**Authors:** Yanlei Yu, Honggang Wang, Xiaoshu Jin, Wenjing Huang, Yunjie Zhao, Ningning Wang, Dongze Lu, Bin Wei, Hong Wang

**Affiliations:** 1 College of Pharmaceutical Science & Collaborative Innovation Center of Yangtze River Delta Region Green Pharmaceuticals, Zhejiang University of Technology, Hangzhou 310014, China; yanleiyu@zjut.edu.cn (Y.Y.); binwei@zjut.edu.cn (B.W.); 2 Binjiang Cyberspace Security Institute of ZJUT, Hangzhou 310056, China

**Keywords:** *Dendrobium officinale* polysaccharide, structural characterization, metabolism regulation, gut microbiota

## Abstract

Polysaccharides derived from *Dendrobium officinale* have been demonstrated to exhibit metabolic regulatory properties. However, the correlation between their structure and function, particularly their mechanism of action through gut microbiota, remains underexplored. This study systematically elucidates the structural characteristics of Dendrobium officinale polysaccharide (DOP) from the Guizhou (GZ) and Zhejiang (ZJ) provinces of China using nuclear magnetic resonance (NMR) and a series of chromatographic analyses, revealing their unique molecular features. Additionally, the metabolic regulatory activities were assessed through α-glucosidase inhibitory assay and in vitro intestinal flora activity assay. The findings include the following: (1) both DOP-GZ and DOP-ZJ predominantly consist of glycosidic linkages of β-1,4-Man*p* and β-1,4-Glc*p*; (2) zhe monosaccharide composition ratios of mannose to glucose are 2.51:1 for DOP-GZ and 2.66:1 for DOP-ZJ, with molecular weights of 356 kDa and 544 kDa, respectively, indicating significant structural differences between DOPs from different sources; (3) treatment with DOP-GZ and DOP-ZJ led to alterations in the α-diversity indices and *Firmicutes*-to-*Bacteroidota* ratios; (4) more importantly, DOP-GZ and DOP-ZJ significantly increase the abundance of beneficial bacteria (e.g., g_Proteobacteria_unclassified) while suppressing the growth of pathogenic bacteria (e.g., f_Enterobacteriaceae_unclassified), with statistically significant results. These findings not only uncover a novel mechanism by which DOPs regulate metabolism through gut microbiota but also provide a crucial theoretical basis for the application of DOPs in functional foods and pharmaceutical development.

## 1. Introduction

*Dendrobium officinale,* a valuable traditional Chinese medicine (TCM), is officially documented in the Chinese Pharmacopoeia [1]. It is predominantly distributed in southern China, particularly in provinces such as Yunnan, Sichuan, Guangxi, and Zhejiang [2]. *Dendrobium officinale* exhibits a wide range of pharmacological properties, including anti-aging [3], antioxidant [4], immunoregulatory [5], and anti-fatigue effects [6]. Additionally, it is utilized for managing conditions like diabetes, obesity, gastrointestinal diseases, and other ailments [7]. The primary active components of *Dendrobium officinale* encompass phenols, alkaloids, terpenes, flavonoids, and amino acids, with polysaccharides being recognized as one of its most critical constituents. Dendrobium officinale polysaccharides (DOPs), classified as acetylated glucomannans, exhibit structural and physicochemical properties that vary depending on their source and extraction methods [8].

Polysaccharides derived from *Dendrobium officinale* have been extensively studied for their impact on metabolic regulation. Excessive calorie consumption has been associated with various health issues such as elevated blood pressure, high blood glucose level, increased body fat, abnormal cholesterol or triglyceride levels, and the development of metabolic disorders. These conditions can escalate the risk of heart disease, stroke, and type 2 diabetes if left unaddressed [9]. Research has shown that DOP can influence lipid, glucose, and protein metabolism, as well as modulate the composition and function of gut microbiota, leading to beneficial outcomes [10]. Administration of DOP orally has been found to introduce components that are indigestible and non-absorbable, thereby affecting gut microbiota [11]. Both in vivo and in vitro studies have demonstrated that DOPs resist digestion and are primarily metabolized by gut microbiota in the large intestine, producing short-chain fatty acids that serve as energy sources, enhance mineral absorption, and impact the diversity and composition of gut microbiota [12]. Previous investigations suggest that DOPs possess the capacity to restore gut microbiota homeostasis and alleviate oxidative stress in glial cells. Dietary interventions, particularly those targeting gut microbiota, have been shown to influence intestinal function [3,13]. Nevertheless, the correlation between the structural properties of DOP and its effects on metabolic regulation through gut microbiota remains incompletely explored.

The proposed structure of DOP has been extensively researched and primarily comprises varying proportions of mannose and glucose to form backbone structures of (1→4)-β-D-Manp, (1→4)-β-D-Glcp, and 2-OAc(1→4)-β-Manp [14]. There has also been research reporting that the backbone of DOP is 1,4-mannan [15,16]. It has been noted that the molecular weight distribution and acetyl group substitutions of DOP are closely linked to their biological activities [17,18]. Previous research has indicated that DOPs exhibit structural differences based on their source, which can include factors such as growth environment, light intensity, and soil composition [19]. TCM often emphasizes the authenticity of medicinal materials, which can be influenced by factors like climate, temperature, humidity, and other environmental conditions [20]. To more effectively explore the relationship between the structure of DOPs and their regulatory effects on metabolism, DOPs from various sources have been analyzed to identify diverse structural characteristics. The fundamental structure of the isolated DOPs was determined through a range of modern analytical techniques, including gel permeation chromatography, high-performance liquid chromatography, Fourier transform infrared spectroscopy, methylation derivatization followed by gas chromatography, and nuclear magnetic resonance. Previous studies conducted by our team have demonstrated that seaweed polysaccharides can mitigate obesity induced by a high-fat diet and enhance the population of carbohydrate-degrading gut bacteria, indicating that these polysaccharides may exert anti-obesity effects by modulating the gut microbiota [21,22].

In the present study, the physicochemical and structural properties of DOPs originating from different sources were systematically evaluated. The regulatory effects of these DOPs on metabolism were further investigated using an α-glucosidase inhibitory assay and an in vitro intestinal flora activity assay. Compared to previous studies, the novelty of this study was to provide new insights into the mechanisms by which DOPs from diverse sources regulate metabolism through interactions with gut microbiota.

## 2. Materials and Methods

### 2.1. Materials and Reagents

*Dendrobium officinale* was provided by Zhejiang Jigongyuan Pharmaceutical Co., Ltd. (Zhejiang, China) and Guizhou Lizeng Agricultural Tourism Development Co., Ltd. (Guizhou, China). Dextrans with different molecular weight were purchased from Aladdin Scientific (Shanghai, China). Monosaccharide standards, including arabinose, fucose, mannose, rhamnose, glucose, galactose, glucuronic acid, and galacturonic acid, were obtained from American Polymer Standards Corporation (Mentor, OH, USA). All reagents utilized in this study were of analytical grade, unless specified otherwise.

### 2.2. Extraction and Purification of DOP

Fresh *D. officinale* stem samples (200 g) were ground into a powder using a grinder and subsequently sieved through a 100-mesh sieve. The sample underwent defatting by the addition of ethanol (1:5, g/mL) and magnetic stirring (400 rpm) for 2 h at room temperature. The resulting residue was obtained through filtration under reduced pressure and then dried in an oven at 60 °C. Polysaccharides were extracted by hot water extraction under optimized conditions. The defatted samples were subjected to hot water extraction at 60 °C for 2 h, repeated 3 times. The filtrates were centrifuged, combined, concentrated, and mixed with 4 times the volume of anhydrous ethanol for precipitation overnight. The precipitate was separated by centrifugation (4000 rpm, 20 min), washed multiple times with ethanol, and dried in an oven to yield the crude polysaccharide. The crude polysaccharide (2 g) was dissolved in 200 mL of deionized water, mixed thoroughly, and centrifuged at 4000 rpm for 25 min at room temperature, retaining the supernatant. This process was repeated through three cycles of freezing and thawing, followed by centrifugation at 4000 rpm for 20 min, and filtration through 0.8 μm and 0.45 μm microporous filtration membranes. The starch was next removed by α-amylase digestion. The supernatant was then dialyzed against a 3 kDa molecular weight cutoff membrane for 48 h and lyophilized to obtain DOP. The remaining starch was determined by 3, 5-dinitrosalicylic acid colorimetric method using glucose as the control [23]. The purity of DOP was assessed using high-performance gel permeation chromatography with dextran as the standard.

### 2.3. Molecular Weight Determination by GPC

The molecular weight distribution of DOPs was determined using GPC-HPLC equipped with a TSKgel G5000PWxl column (Tosoh Corporation, Tokyo, Japan) and an evaporative light scattering detector (ELSD) (Waters Corporation, Milford, CT, USA). The mobile phase was 5 mM ammonium acetate, and isocratic elution was employed with a flow rate of 0.5 mL/min. The sample injection volume was 20 μL. Dextrans of different molecular weights were used as standards for calibration.

### 2.4. Monosaccharide Composition Analysis

The monosaccharide composition of DOP was analyzed using a LC-MS method with slight modifications [24]. Briefly, DOP (2 mg) was hydrolyzed in 2 mol/L trifluoroacetic acid (TFA) at 110 °C for 6 h. Subsequently, methanol (0.2 mL) was added, and the sample was dried three times with a nitrogen blower to eliminate any remaining TFA. Following this, 0.5 M 1-phenyl-3-methyl-5-pyrazolone (PMP) was introduced to the acid hydrolysate for derivatization at 70 °C for 30 min. The pH was then adjusted to neutral with HCl, and chloroform was utilized to eliminate any residual PMP derivatizing agent. The resulting supernatant was filtered through a 0.22 μM membrane and subjected to analysis using an ExionLC system coupled with a TripleQuad mass spectrometer operating in multiple reaction monitoring (MRM) mode (AB SCIEX, Framingham, MA, USA). An ACQUITY Premier HSS T3 column (1.8 μm, 2.1 × 100 mm) (Waters Corporation, Milford, CT, USA) was utilized for the separation, with a flow rate set at 0.3 mL/min. The monosaccharide composition was determined through isocratic elution of acetonitrile and deionized water containing 5 mM ammonium acetate in a ratio of 75:25 (*v*/*v*).

### 2.5. Scanning Electron Microscope (SEM) Analysis

DOP powders were applied to the surface of a double-sided adhesive tape, ensuring the formation of a uniform thin layer. Subsequently, the samples were placed in a vacuum coater for gold sputtering and analyzed using a scanning electron microscope (SEM, Hitachi, S-4700, Tokyo, Japan) under vacuum conditions at an accelerating voltage of 10 kV.

### 2.6. Fourier Transform-Infrared (FT-IR) Analysis

The lyophilized DOP samples (2 mg) were compressed into a circular form using the potassium bromate tablet preparation technique and analyzed with a Fourier transform infrared (FT-IR) spectrometer within the frequency range of 4000–400 cm^−1^ (Thermo Fisher Scientific, Waltham, MA, USA).

### 2.7. Glycosidic Linkage Analysis by Methylation

Dried DOP (10 mg) was mixed with 5 mL of dimethyl sulfoxide. The container was purged with argon to establish an environment free of moisture and oxygen. The mixture was stirred with a magnetic stirrer until it was completely dissolved. Powdered sodium hydroxide was added to the aqueous solution and allowed to undergo a reaction within a sealed container immersed in an ice water bath for 2 h. Subsequently, 1 mL of iodomethane was injected using a syringe in the absence of light and allowed to react for an additional 2 h. The reaction was terminated by the addition of 2 mL of water. The methylated polysaccharides were extracted by vigorously shaking the solution three times with an equal volume of chloroform. The chloroform layer was collected, dehydrated through centrifugation, and transferred to a vial. Subsequently, 1 mL of 90% formic acid was added to the vial and subjected to hydrolysis at 100 °C for 6 h. Following the addition of 1 mL of methanol in each step, the formic acid was evaporated using nitrogen gas, repeated three times. Next, 4 mL of 2 mol/L trifluoroacetic acid was added and allowed to react at 100 °C for 6 h. The trifluoroacetic acid was eliminated using methanol, and the resulting product was dissolved in 3 mL of water. A quantity of 40 mg of sodium borohydride was added to the container and allowed to react for 3 h at room temperature, then neutralized with acetic acid and dried. Subsequently, 2 mL of acetic anhydride and pyridine were added separately, and the reaction was carried out for 1 h at 100 °C, followed by termination with 1 mL of water. The derivative was extracted with chloroform three times and was collected using a rotary evaporator for subsequent GC-MS analysis.

### 2.8. One-Dimensional and Two-Dimensional NMR Spectroscopy Analysis

DOPs were dissolved in 0.4 mL D_2_O and lyophilized, repeated twice. Subsequently, 1D (^1^H, ^13^C) and 2D (COSY, HSQC) NMR spectroscopy analyses were performed on a Bruker Avance 500 MHz spectrometer (Bruker, Billerica, MA, USA).

### 2.9. α-Glucosidase Inhibition Assay

The α-glucosidase inhibitory activity of DOPs was assessed following the protocol outlined by Matsui et al., with some modifications [25]. Briefly, 10 μL of DOP samples (6.7 mg/mL) were added to 25 μL of α-glucosidase and 140 μL of phosphate buffer, followed by an incubation at 37 °C for 10 min. Subsequently, 25 μL of *p*NPG (24 mmol/L) was added and the sample was allowed to incubate for an addition 15 min, after which the absorbance was recorded at 405 nm. Acarbose was used as the positive control.

### 2.10. Gut Microbiota Analysis

Normal anaerobic broth medium underwent autoclaving at 121 °C for 20 min and was then equilibrated in an anaerobic workstation for 3 days. Healthy mouse feces were obtained by adding PBS at a ratio of 1:10 (*w*/*v*), agitating for 5 min, and subsequently centrifuging at 500 r/min at 4 °C for 5 min. The resulting supernatant was further centrifuged at 15,000 r/min at 4 °C for 20 min, yielding a sediment at the bottom. DOPs were re-dissolved in an aqueous solution at a concentration of 6.7 mg/mL. The subsequent procedures were performed in a sterilized anaerobic workstation. Specifically, 1 mL of DOP was added to 1 mL of bacterial solution and 6 mL of medium, followed by incubation for 1 week. A control group was established using 1 mL of deionized water, with each group undergoing three parallel experiments. The sedimented bacteria were isolated through centrifugation at 15,000 r/min for 20 min at 4 °C and subsequently subjected to sequencing analysis. The microbial DNA was extracted from cecal content using a KAPA HiFi HotStart Ready Mix Kit. Bioinformatic analysis of the sequencing data was conducted on the microbiome analysis platform QIIME2 2022.2 (Quantitative Insights Into Microbial Ecology, USA).

### 2.11. Statistical Analysis

In this study, GraphPad Prism software (version 8.0) was employed for data analysis. A one-way analysis of variance (ANOVA) was conducted to test differences among groups, while a *t*-test was utilized to determine the statistical significance of differences between two experimental groups (*p* < 0.05).

## 3. Results and Discussion

### 3.1. Isolation and Purification of DOP

The crude polysaccharides were extracted through a series of processes involving ethanol defatting, hot water extraction, and repeated ethanol precipitation. Subsequently, the polysaccharides underwent purification using a repeated freeze–thaw combined with starch-removing method. In this procedure, the polysaccharides were subjected to freezing at −20 °C for 6 h, following by thawing at 35 °C. The cycle was repeated three times, and the resulting supernatant was filtered sequentially through 0.8 μm and 0.45 μm microporous membranes; subsequently, the samples underwent α-amylase hydrolysis. Following dialysis and lyophilization of the supernatant, purified polysaccharides from *Dendrobium officinale*, specifically identified as DOP-GZ and DOP-ZJ, were obtained. The purity of DOP was assessed using an extrinsic method that involved creating a standard curve with dextran, revealing a purity of 91.3% for DOP-GZ and 89.4% for DOP-ZJ, respectively. The starch content was reduced from 1.86% to 0.22% for DOP-GZ and from 1.68% to 0.40% for DOP-ZJ. The physicochemical characteristics of both DOP-GZ and DOP-ZJ are presented in Table 1, demonstrating high purity and low protein content for both variants.

### 3.2. Molecular Weight Determination and Monosaccharide Composition Analysis

**Figure 1:**
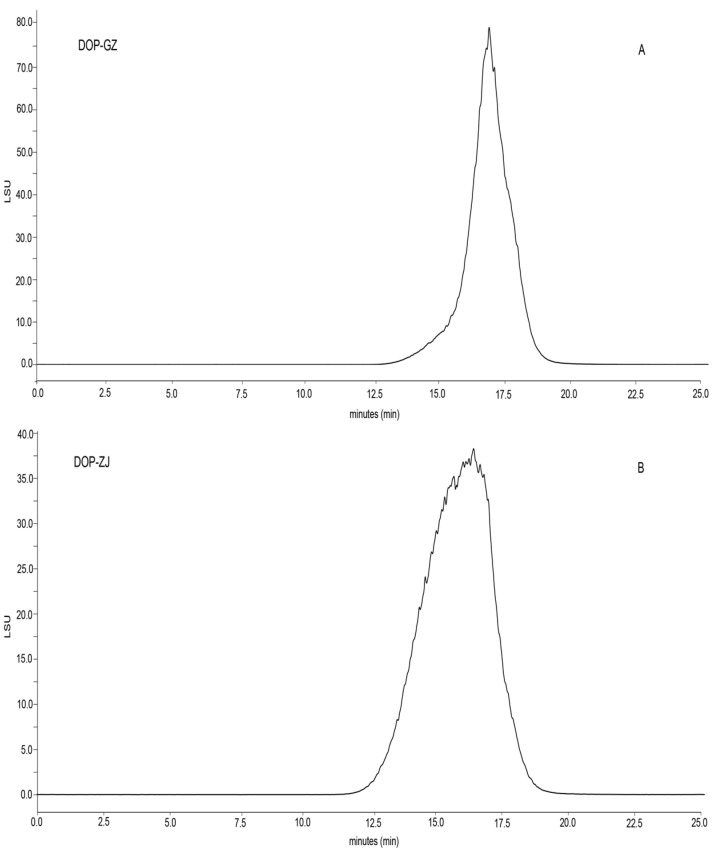
Molecular weight distribution of DOP-GZ and DOP-ZJ: (**A**) DOP-GZ; (**B**) DOP-ZJ.

**Figure 2:**
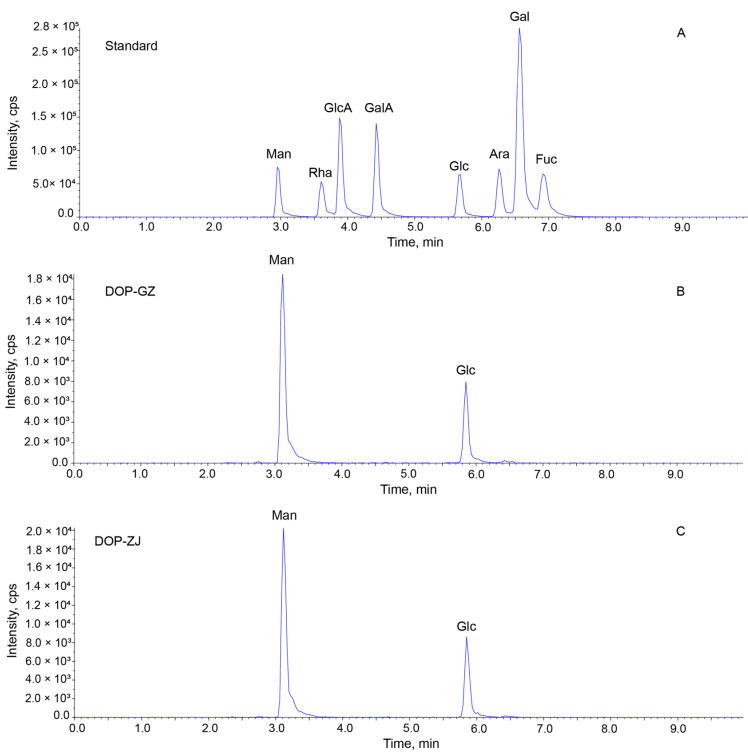
Monosaccharide analysis of DOP-GZ and DOP-ZJ: (**A**) monosaccharide standards; (**B**) DOP-GZ; (**C**) DOP-ZJ.

HPLC-GPC is a highly effective technique for determining the molecular weight of polysaccharides [26]. The results showed that the peak shape of DOP-GZ was narrow and sharper, whereas the peak shape of DOP-ZJ exhibited more symmetry (Figure 1). The distribution of molecular weight is recognized as a critical factors influencing the bioactivities of polysaccharides [27]. By utilizing the average molecular weight of dextran as a reference standard, the molecular weights of DOP-GZ and DOP-ZJ were determined to be 356 kDa and 544 kDa, respectively. Monosaccharide serves as the fundamental building blocks of polysaccharide, impacting both bioactivity and detectability due to their simplicity [28]. Analysis of monosaccharide composition revealed that both DOP-GZ and DOP-ZJ consist of Man and Clc (Figure 2). The molar ratios of Man to Glc in DOP-GZ and DOP-ZJ were found to be 2.51:1 and 2.66:1, respectively (Table 1).

### 3.3. SEM Analysis

**Figure 3:**
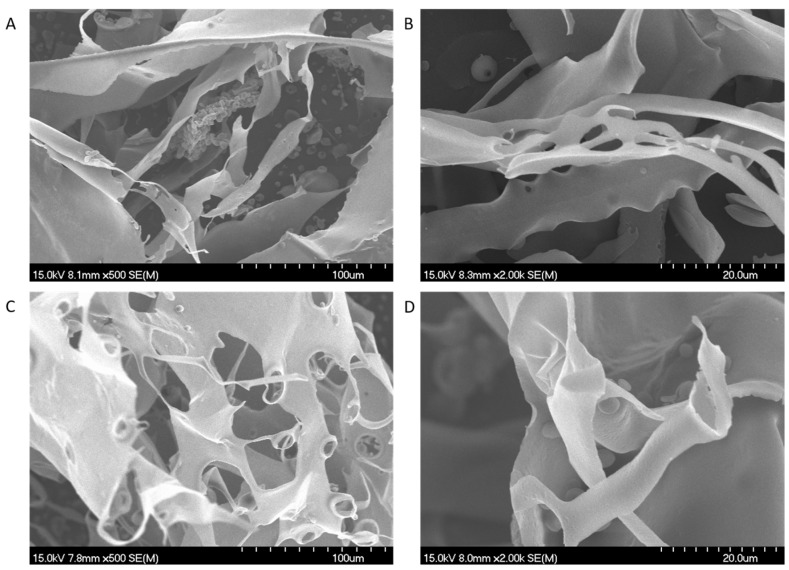
Scanning electron microscope images of DOP-GZ and DOP-ZJ: (**A**) DOP-GZ ×500; (**B**) DOP-GZ ×2000; (**C**) DOP-ZJ×500; (**D**) DOP-ZJ ×2000.

A scanning electron microscope (SEM) was mainly applied for observing the morphology and surface structure of the sample [29]. The results of SEM of DOP-GZ and DOP-ZJ are shown in Figure 3. The results showed that the particles of DOPs were dense, aggregated, irregular in shape, and had different degrees of wrinkles on the surface.

### 3.4. FT-IR Spectroscopy

**Figure 4:**
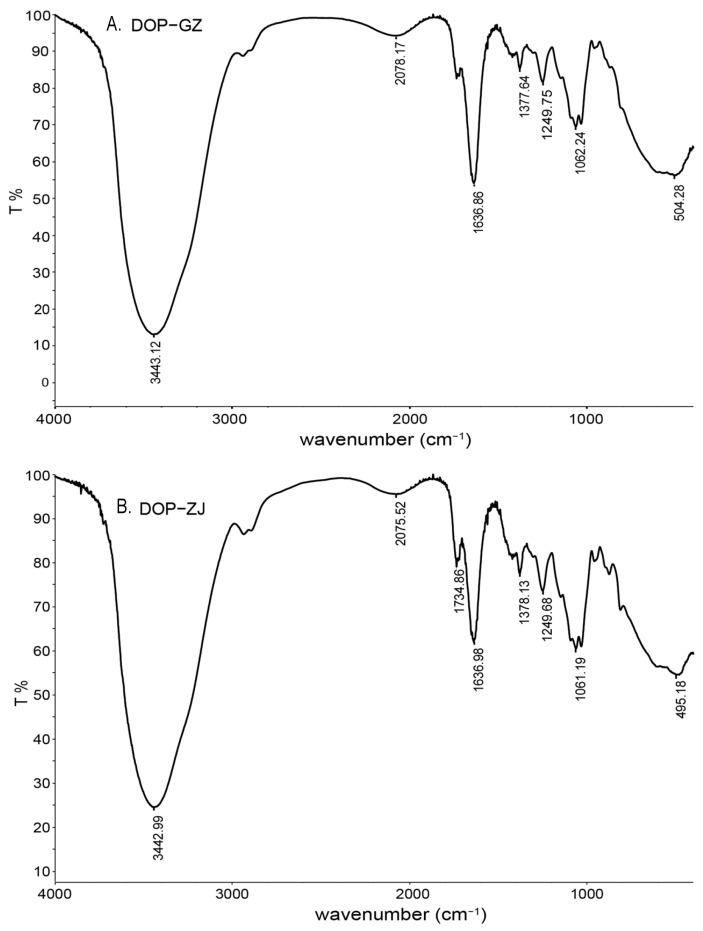
FT-IR analysis of DOP-GZ and DOP-ZJ: (**A**) DOP-GZ; (**B**) DOP-ZJ.

The infrared spectra of DOP-GZ and DOP-ZJ showed various typical absorption bands characteristic of polysaccharides (Figure 4). The prominent and broad bands observed at 3443 cm^−1^ for DOP-GZ and 3442 cm^−1^ for DOP-ZJ were attributed to the OH stretching vibrations of hydroxyl groups. The bands at 1637 cm^−1^, 1377 cm^−1^, and 1062 cm^−1^ for DOP-GZ and 1637 cm^−1^, 1378 cm^−1^, and 1061 cm^−1^ for DOP-ZJ represent the C=O, CH, and C-O vibrations of carboxyl groups (-COOCH_3_), suggesting the presence of acetyl groups in both DOP-ZJ and DOP-GZ. The absorption peaks observed at 1734 cm^−1^ for both DOP-ZJ and DOP-GZ indicate the presence of C=O stretching vibrations of carboxyl groups (-COO-), suggesting the existence of uronic acid. The three peaks at 1150 cm^−1^, 1062 cm^−1^, and 1029 cm^−1^ for DOP-GZ and 1150 cm^−1^, 1061 cm^−1^, and 1029 cm^−1^ for DOP-ZJ are associated with the stretching vibrations of the pyranose ring. Additionally, the absorptions at 874 cm^−1^ and 809 cm^−1^ for DOP-GZ and 875 cm^−1^ and 810 cm^−1^ for DOP-ZJ indicate a β-dominant configuration in mannose and glucose. These findings provide evidence that DOP-GZ and DOP-ZJ are polysaccharides composed of glucose and mannose through a β-pyranose ring [30].

### 3.5. Glycosidic Linkage by Methylation Analysis

A conventional methylation analysis was carried out on both DOP-GZ and DOP-ZJ to elucidate the glycosidic linkages and branching patterns of the polysaccharides using the Need and Selvenran method [31]. This was followed by hydrolysis, reduction, and acetylation to produce alditol acetates. The obtained GC-MS spectra were compared against the database available at the Complex Carbohydrate Research Center (https://glygen.ccrc.uga.edu/ccrc/specdb/ms/pmaa/pframe.html, accessed on 6 August 2024). The findings indicated that the primary linkage types present in both DOP-GZ and DOP-ZJ were 1-4-Man*p*, with a relative abundance of 61.0% and 56.5%, respectively, along with smaller quantities of 1-4-Glc*p*, 3,4-Man*p*, 4,6-Man*p*, and terminal-Man*p* (Table 2). These results suggested that both DOP-GZ and DOP-ZJ exhibited a backbone composed of 1,4-linked mannose and 1,4-linked glucose, as well as mannose branching, which aligned with previous studies [14].

### 3.6. NMR Analysis

**Figure 5:**
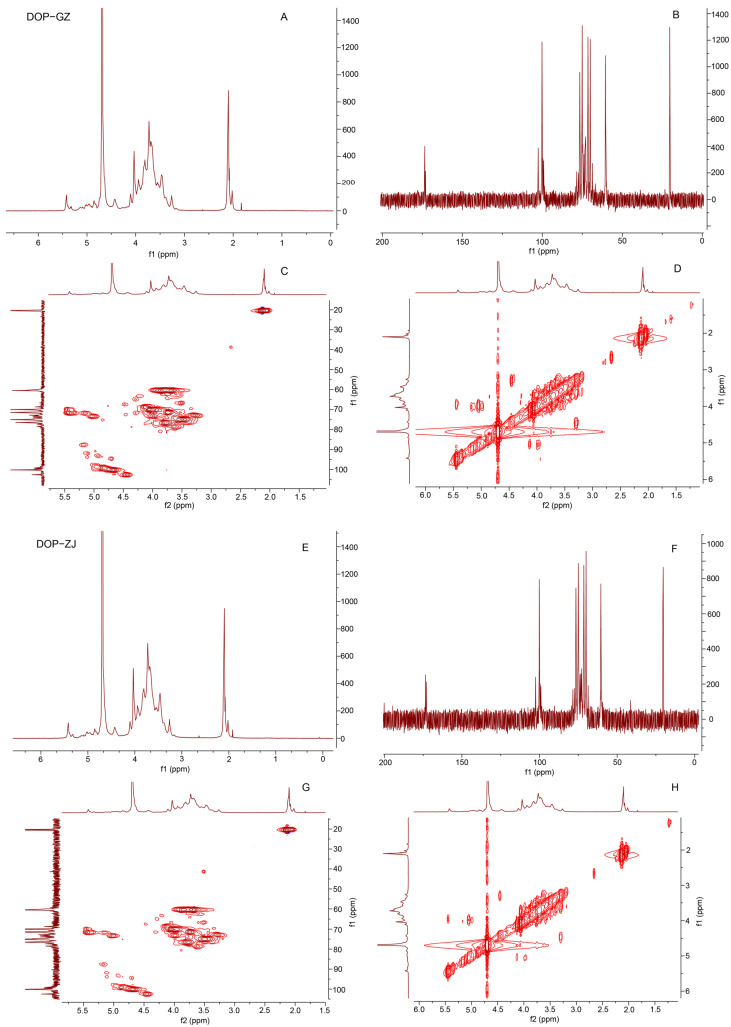
1D and 2D NMR of DOP-GZ and DOP-ZJ: (**A**) ^1^H of DOP-GZ; (**B**) ^13^C of DOP-GZ; (**C**) HSQC of DOP-GZ; (**D**) COSY of DOP-GZ; (**E**) ^1^H of DOP-ZJ; (**F**) ^13^C of DOP-ZJ; (**G**) HSQC of DOP-ZJ; (**H**) COSY of DOP-ZJ.

NMR spectroscopy can provide comprehensive structural information, such as linkage patterns, anomeric configurations, and monosaccharide compositions. In this study, the structural characteristics of DOP-GZ and DOP-ZJ were identified by 1D-NMR (^1^H and ^13^C) and 2D-NMR (HSQC and COSY) spectra (Figure 5). The ^1^H-NMR signals of DOP-GZ and DOP-ZJ predominantly appeared within the 2.0~5.5 ppm range, with a D_2_O signal at 4.7 ppm, and the 3.2–4.2 ppm was due to the stacking of proton signals on the sugar ring, while the major end-base proton peaks were distributed centrally in the region of 4.3–5.5 ppm (Figure 5A,E). A signal at 5.44 or 5.45 ppm observed in both DOP-GZ and DOP-ZJ represents the hydrogen in the skeleton of a sugar connected to an acetoxy group. In addition, the signals from 1.9 to 2.2 ppm should be assigned to the hydrogen of the methyl of the acetoxy group. It can be speculated that both DOP-GZ and DOP-ZJ contain an acetoxy group. The chemical shifts observed at 4.69 ppm and 4.45 ppm in ^1^H NMR were attributed to the anomeric peaks of mannose and glucose in DOP-GZ and DOP-ZJ, respectively. Similarly, the carbon spectrum signals were primarily clustered between 60 and 120 ppm in the ^13^C NMR (Figure 5B,F). Moreover, the chemical shift signals at 2 ppm in the ^1^H NMR, 173 ppm for the carbonyl group (C=O), and 20 ppm for the methyl group (-CH_3_) in the ^13^C NMR confirmed the presence of acetyl groups in the structures of DOP-GZ and DOP-ZJ [32].

Apart from 1D NMR spectroscopy, 2D NMR (^1^H-^1^H COSY and ^1^H-^13^C HSQC) was also applied (Figure 5C,D,G,H). The attribution of the NMR signals was based on published papers and structural elucidation experience [13,33]. According to the 1D NMR spectrum, five cross peaks were found in the HSQC spectrum (Figure 5C,G) at 4.45/102.50, 4.69/100.21, 4.88/99.13, and 4.96/98.48 ppm for DOP-GZ and 4.45/102.55, 4.69/100.14, 4.88/99.11, 4.93/93.27, and 4.99/99.56 ppm for DOP-ZJ, which indicated that DOP-GZ and DOP-ZJ may consist of five sugar residues. The H2 of →4)-β-Glc*p*-(1→ was jointly determined by the cross peaks at 4.45/3.29 ppm in ^1^H-^1^H COSY and 3.28/72.89 ppm in ^1^H-^13^C HSQC for DOP-GZ, respectively. The presence of H2–H6 was indicated by the cross peaks at 3.28/3.29, 3.29/3.64, 3.64/3.49, and 3.49/3.85 ppm in the COSY spectrum. The carbon shifts can be assigned by the ^1^H-^13^C HSQC spectrum. Previous studies have reported significant differences in the C/H signals associated with the α/β configurations of reduced-end glucose. In ^1^H NMR spectra, the chemical shift associated with the α-configuration is typically greater than 4.9 ppm, whereas the β-configuration exhibits the opposite trend. In ^13^C NMR spectra, signals corresponding to the β-configuration are generally observed at values exceeding 100 ppm, in contrast to those of the α-configuration. The residue was identified as →4)-β-Glc*p*-(1→, which was consistent with the methylation analysis and previously reported results [34]. Similarly, based on the COSY association of H1–H6 and HSQC, the main chain was considered to be →4)-β-Manp-(1→ on the basis of methylation and spectral results. Furthermore, due to the significant enhancement of the chemical shifts at H2 and H3, combined with the results of FT-IR, it was demonstrated that the chain contained the –O-acetyl group, located at the O-2 position. Further analysis of the COSY and HSQC spectra allowed for the identification of the remaining signals, as detailed in Table 3.

**Figure 6:**
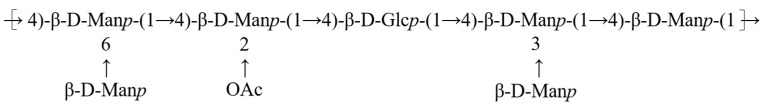
Presumed overall structure of DOP.

The presumed overall structure of DOP is presented in Figure 6.

### 3.7. Inhibitory Effects of DOPs on α-Glucosidase

The inhibition assays for α-glucosidase were used to assess the antidiabetic properties of DOP-GZ and DOP-ZJ. α-Glucosidase is a key enzyme involved in carbohydrate digestion, facilitating the hydrolysis of the 1,4-α bonds in non-absorbed oligosaccharides, converting them into glucose. Compounds that inhibit α-glucosidase can impede the breakdown and absorption of dietary carbohydrates, thus hindering glucose uptake into the bloodstream [35,36]. Comparative analysis with acarbose, a standard reference drug, revealed that both DOP-GZ and DOP-ZJ demonstrate α-glucosidase inhibition activity, but lower than acarbose, as shown in Table 4, indicating their potential effectiveness in reducing blood glucose levels.

### 3.8. Gut Microbial Analysis

**Figure 7:**
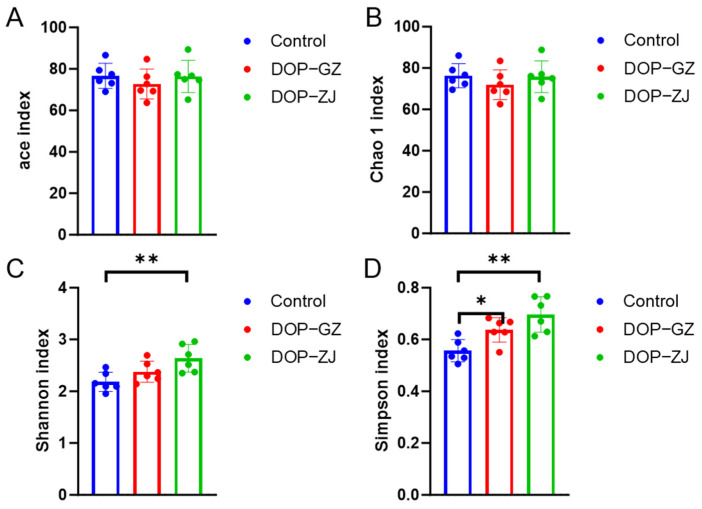
Effects of DOP-ZJ and DOP-GZ on α-diversity of gut microbiota in vitro fermentation experiments. (**A**) ACE index, (**B**) Chao 1 index, (**C**) Shannon index, (**D**) Simpson index. *, *p* < 0.05, **, *p* < 0.01.

The impact of DOP treatment on the composition of gut microbiota was assessed through 16S rRNA gene sequencing. Polysaccharides that are indigestible have been shown to modulate the composition and function of gut microbiota, thereby exerting significant influence on metabolic regulation [11,37]. α-Diversity refers to the analysis of species diversity within a single sample, which serves as an indicator of the richness and diversity of the microbial community [38]. Community richness can be quantified using the Chao and ACE indices, while community diversity is evaluated using the Shannon and Simpson indices. Higher values of the ACE, Chao 1, and Shannon indices indicate greater community diversity, whereas higher Simpson index value suggests lower community diversity [39]. A comparison with the control group indicated an increase in the Shannon index and a decrease in the Simpson index in both the DOP-ZJ and DOP-GZ groups (Figure 7C,D). In contrast, no significant changes were observed in the ACE and Chao 1 indices (Figure 7A,B). These findings suggest that DOP treatment may enhance community diversity without affecting community richness. A significant increase in the Shannon diversity index (*p* < 0.01) and a decrease in the Simpson index (*p* < 0.01) were observed in the DOP-ZJ group. Additionally, a significant decrease in the Simpson index was noted in the DOP-GZ group (*p* < 0.05), indicating that DOP-ZJ had a more pronounced effect on altering microbial composition. Previous studies have demonstrated a close association between the Shannon diversity index and the phyla *Bacteroidota* and *Firmicutes* [40]. These findings suggest that DOPs may modulate metabolic processes by enhancing α-diversity, as evidenced by the changes in the Shannon index.

**Figure 8:**
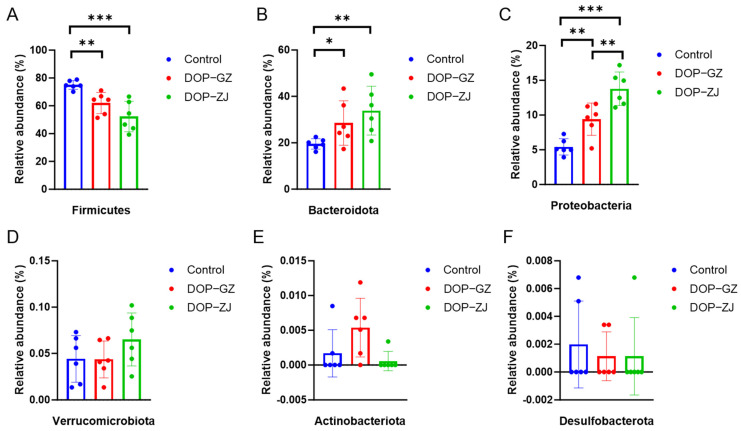
Relative abundance of gut microbiota at the phylum level in vitro fermentation experiments: (**A**) Firmicutes; (**B**) Bacteroidota; (**C**) Proteobacteria; (**D**) Verrucomicrobiota; (**E**) Actinobacteriota; (**F**) Desulfobacterota. *, *p* < 0.05, **, *p* < 0.01, ***, *p* < 0.001.

Subsequently, this study examined the effects of DOP treatment on gut microbiota at the phylum level in the feces of mice. The predominant phyla identified were *Firmicutes, Bacteroidota, and Proteobacteria*, which collectively accounted for 99% of the total bacterial population. In contrast, *Verrucomicrobiota*, *Desulfobacterota*, and *Actinobacteriota* were found to be present in low abundances (Figure 8 and Figure S1). Notably, the abundance of *Firmicutes* in the DOP-treated group decreased significantly, with DOP-GZ exhibiting a significant difference (*p* < 0.01) and DOP-ZJ showing an even more pronounced reduction (*p* < 0.001) (Figure 8A). Conversely, the abundance of *Bacteroidota* and *Proteobacteria* increased significantly, with DOP-GZ demonstrating significant differences in both phyla (*p* < 0.05 for *Bacteroidota* and *p* < 0.01 for *Proteobacteria*), while DOP-ZJ exhibit even more significant differences (*p* < 0.05 for *Bacteroidota* and *p* < 0.01 for *Proteobacteria*) (Figure 8B,C). The remaining phyla did not show significant differences (Figure 8D–F). Previous research has indicated that both *Firmicutes* and *Bacteroidota* rely on carbohydrate-active enzymes for the digestion of polysaccharide [41]. Numerous studies have reported that individuals with obesity or models induced by a high-fat diet typically exhibit an increase in the relative abundance of *Firmicutes* alongside a decrease in *Bacteroidota*, resulting in a higher *Firmicutes*-to-*Bacteroidota* (F/B) ratios [42]. Elevated F/B ratios are often associated with obesity and metabolic disorders, which may be linked to increased caloric extraction from food, fat deposition, adipogenesis, and impaired insulin sensitivity [43]. Furthermore, the relative abundance of *Proteobacteria* demonstrated a significant increase between DOP-GZ and DOP-ZJ groups (*p* < 0.01). Our findings are consistent with previous research suggesting that polysaccharides can influence body weight by altering the *Firmicutes*-to-*Bacteroidota* ratios. The correlation analysis revealed a strong association between the dominant phyla and metabolic regulation, indicating a potential target for intervention.

**Figure 9:**
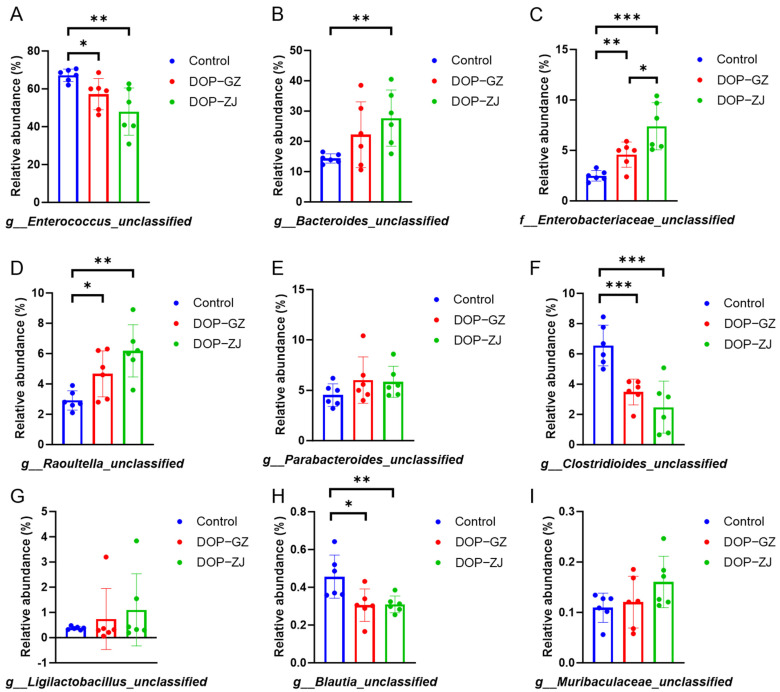
The relative abundance of gut microbiota at species level: (**A**) g__Enterococcus_unclassified; (**B**) g__Bacteroides_unclassified; (**C**) f__Enterobacteriaceae_unclassified; (**D**) g__Raoultella_unclassified; (**E**) g__Parabacteroides_unclassified; (**F**) g__Clostridioides_unclassified; (**G**) g__Ligilactobacillus_unclassified; (**H**) g__Blautia_unclassfied; (**I**) g_Muribaculaceae_unclassified. *, *p* < 0.05, **, *p* < 0.01, ***, *p* < 0.001.

The influence of DOPs on the abundance of microbial communities at the species level has demonstrated significant alterations in the functional gene composition of the microbiota in fecal samples treated with DOP-GZ and DOP-ZJ. Principal component analysis indicates that the DOP treatment groups exhibited substantial differences when compared to the control group (Figure S2). Notably, the species *g__Enterococcus_unclassifed*, *g__Clostridioides_unclassified*, and *g__Blautia_unclassified* experienced significant reductions relative to the control group (Figure 9A,F,H). Specifically, DOP-GZ and DOP-ZJ treatments results in significant differences in *g__Enterococcus_unclassifed* and *g__Blautia_unclassified* (*p* < 0.05 and *p* < 0.01, respectively), with even more pronounced changes observed in *g__Clostridioides_unclassified* (both DOP-GZ and DOP-ZJ, *p* < 0.001). Conversely, *g__Bacteroides_unclassified*, *f__Enterobacteriaceae_unclassified*, and *g__Raoultella_unclassified* exhibited significant increases (Figure 9B–D). Furthermore, *f__Enterobacteriaceae_unclassified* demonstrated a significant increase between DOP-GZ and DOP-ZJ (*p* < 0.05). Meanwhile, *g__Parabacteroides_unclassified*, *g__Ligilactobacillus_unclassified*, and *g__Muribaculaceae_unclassified* showed variations in abundance but did not reach statistical significance (Figure 9E,G,I). In terms of decreasing species, *g__Clostridioides_unclassified* is a bacterium that can cause severe intestinal illness; DOP-GZ and DOP-ZJ treatment can inhibit the growth of this bacterium [44]. In contrast, the increasing species *g__Bacteroides_unclassified* is recognized as a beneficial bacterium for humans and plays a crucial role in the digestion of dietary fiber polysaccharides and host glycans. The polysaccharide utilization sites of Anabaena serve as a significant protein mechanism for the acquisition of various carbohydrates and the initiation of metabolic processes [45]. Although *g__Muribaculaceae_unclassified* showed a slight increase, it did not exhibit significant differences. This species is considered a potential probiotic that may beneficially mitigate metabolic syndrome by producing short-chain fatty acids through the fermentation of endogenous fucosylated glycans or exogenous polysaccharides, thus representing a promising area for further research [46].

### 3.9. Structure-Activity Relationships

The metabolic regulatory effects of DOPs exhibit variability based on their structural characteristics, including Mw distribution, monosaccharide composition, and modifications. Previous studies have indicated a positive correlation between Mw and metabolic regulation activities, while the ratio of mannose to glucose demonstrated an inverse relationship [47]. The degree of acetylation displayed a negative correlation with renal and hepatic function, impacting glucose and lipid metabolism [48]. Our findings revealed that both DOPs contain varying proportions of 1,4-glycosidic-linked mannose and glucose, with DOP-GZ having 61.0% and DOP-ZJ having 56.5% of 1,4-linked mannose. Additionally, DOP-ZJ exhibited more side chains, including 3,4-linked mannose and 4,6-linked mannose, compared to DOP-GZ. It has been reported that neutral monosaccharides in the side chain of polysaccharides are preferentially utilized [49]. This may explain why DOP-ZJ demonstrated superior metabolic regulatory activity compared to DOP-GZ. Studies have shown that a high mannose content can reduce intestinal microbiota utilization due to the rates and types of metabolites [50]. The mannose to glucose ratio was 2.51:1 and 2.66:1 in DOP-GZ and DOP-ZJ, respectively, in this study, aligning with previous reports. Molecular weight is another critical factor influencing biological activities. Generally, polysaccharides with higher Mw exhibit increased activities; however, high Mw polysaccharide often have poor solubility, limiting their efficacy. In our study, DOP-ZJ with a higher Mw demonstrated better activity than DOP-GZ. The presence of acetyl groups is also closely linked to biological activities. NMR analysis identified acetyl modifications in both DOP-GZ and DOP-ZJ. However, the quantification of acetyl groups requires further investigation to understand their impact. These results indicate that the metabolic regulatory activities of DOPs are influenced by their structural characteristics. Nonetheless, in vivo animal studies were necessary for the subsequent analysis.

## 4. Conclusions

In this study, the structure and metabolism regulation activities of *Dendrobium officinale* polysaccharides sourced from Guizhou (DOP-GZ) and Zhejiang (DOP-ZJ) provinces were investigated. Structural analysis revealed that both DOP-GZ and DOP-ZJ share a backbone of 1,4-Glcp and 1,4-Manp, with branched chains of 3,4-Manp and 4,6-Manp in varying proportions. However, differences were observed in molecular weight (DOP-GZ: 356 kDa; DOP-ZJ: 544 kDa) and monosaccharide composition (mannose-to-glucose ratio: 2.51:1 for DOP-GZ; 2.66:1 for DOP-ZJ). Both DOP-GZ and DOP-ZJ exhibited α-glucosidase inhibitory activity. In vitro intestinal flora assays showed that DOP-GZ and DOP-ZJ reduced the relative abundance of *Firmicutes* while increasing *Bacteroidota* at the phylum level, altering the *Firmicutes*-to-*Bacteroidota* ratio. At the species level, DOPs modulated the relative abundance of *f_Enterobacteriaceae_unclassified* and *g_Proteobacteria_unclassified*. These findings highlight the fact that structural and functional differences between DOP-GZ and DOP-ZJ are influenced by their production source, underscoring the importance of origin in determining the bioactivity of *Dendrobium* polysaccharides.

## Figures and Tables

**Table 1:** Molecular weight distribution and monosaccharide composition of DOP-GZ and DOP-ZJ.

Sample	Purity (%)	Protein (%)	Starch (%)	Uronic Acid (%)	Mw (kDa)	Monosaccharide (Molar Ratio)	
						Man	Glc
DOP-GZ	91.3	1.45	0.21	2.31	356	2.51	1
DOP-ZJ	89.4	1.54	0.40	2.44	544	2.66	1

**Table 2:** Methylation analysis of DOP-GZ and DOP-ZJ.

Reside Linkage	Retention Time (min)	Detected Mass Fraction (*m*/*z*)	Relative Content (%)	
			DOP-GZ	DOP-ZJ
Terminal-Manp	18.87	43, 57, 71, 87, 101, 117, 129, 145, 161, 191, 205	11.5	8.6
1,4-linked Manp	20.64	43, 45,71, 87, 99, 101, 113, 117, 129, 131, 143, 161, 173, 203, 233	61.0	56.5
1,4-linked Glcp	20.75	43, 45, 71, 87, 99, 101, 113, 117, 129, 131, 143, 161, 173, 203, 233	16.7	19.1
3,4-linked Manp	21.86	43, 58, 87, 97, 117, 129, 149, 172, 185, 203, 231, 305	1.0	2.6
4,6-linked Manp	22.00	43, 71, 85, 101, 117, 127, 142, 161, 171, 187, 201, 231, 261	9.8	13.2

**Table 3:** ^1^H and ^13^C NMR chemical shifts of DOP-GZ and DOP-ZJ.

Glycosyl Residue (DOP-GZ)	H1/C1	H2/C2	H3/C3	H4/C4	H5/C5	H6/C6
→4)-β-Glc*p*-(1→	4.45/102.50	3.28/72.89	3.29/72.85	3.64/78.60	3.49/75.01	3.85/60.43
→4)-β-Man*p*-(1→	4.69/100.21	4.06/70.05	3.75/71.45	3.76/76.59	3.49/75.01	3.69/60.46
→4)-2-O-acetyl-β-Man*p*-(1→	4.88/99.13	5.45/71.59	3.95/73.19	3.79/76.66	3.57/74.85	3.80/60.25
→4,6)-β-Man-(1→	4.93/93.27	3.85/70.48	3.51/66.70	3.42/69.87	3.89/71.02	3.60/65.21
→4,3)-β-Man-(1→	4.96/98.48	3.99/71.69	3.98/73.17	3.79/75.28	3.56/74.00	3.62/60.21
**Glycosyl residue (DOP-ZJ)**	**H1/C1**	**H2/C2**	**H3/C3**	**H4/C4**	**H5/C5**	**H6/C6**
→4)-β-Glc*p*-(1→	4.45/102.55	3.28/72.89	3.29/72.83	3.63/78.70	3.49/75.00	3.85/60.43
→4)-β-Man*p*-(1→	4.69/100.14	4.06/69.98	3.75/71.46	3.76/76.57	3.51/75.00	3.69/60.45
→4)-2-O-acetyl-β-Man*p*-(1→	4.88/99.11	5.44/71.67	3.96/73.20	3.75/76.59	3.53/75.00	3.80/60.12
→4,6)-β-Man-(1→	4.93/93.27	3.86/70.50	3.52/66.78	3.42/70.01	3.90/71.01	3.60/65.31
→4,3)-β-Man-(1→	4.99/99.56	3.99/71.69	3.98/73.17	3.80/75.00	3.57/73.91	3.60/60.52

**Table 4:** α- Glucosidase inhibition activities of DOP-GZ and DOP-ZJ.

Groups	Average Inhibition Rate (%)
Acarbose	98.2 ± 1.0
DOP-GZ	41.5 ± 2.4
DOP-ZJ	57.3 ±5.9

## Data Availability

Data available on request from the authors.

## Supplementary Materials

The following supporting information can be downloaded at https://www.mdpi.com/article/10.3390/polym17060727/s1, Figure S1: effects of DOP-GZ and DOP-ZJ on gut microbiota at the phylum level in vitro fermentation experiments. Figure S2: PCA diagram of principal component analysis at the species level.
