# Supplementary material for: Structural Characterization of Dendrobium officinale Polysaccharides and Their Regulation Effect on Intestinal Microbiota During In Vitro Fermentation
Source: Polymers (Basel). 2025 Mar 10;17(6):727. doi: 10.3390/polym17060727 (PMC11944827; doi:10.3390/polym17060727)
Supplement: Supplementary file 1 [file polymers-17-00727-s001.zip › polymers-3498025-supplementary.pdf]

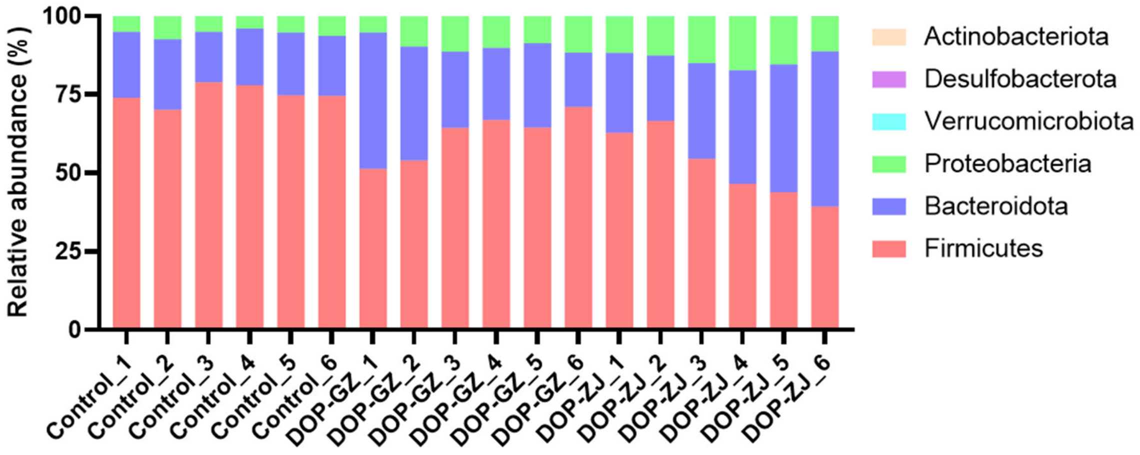

Figure S1. Effects of DOP-GZ and DOP-ZJ on gut microbiota at the phylum level in vitro fermentation experiments.

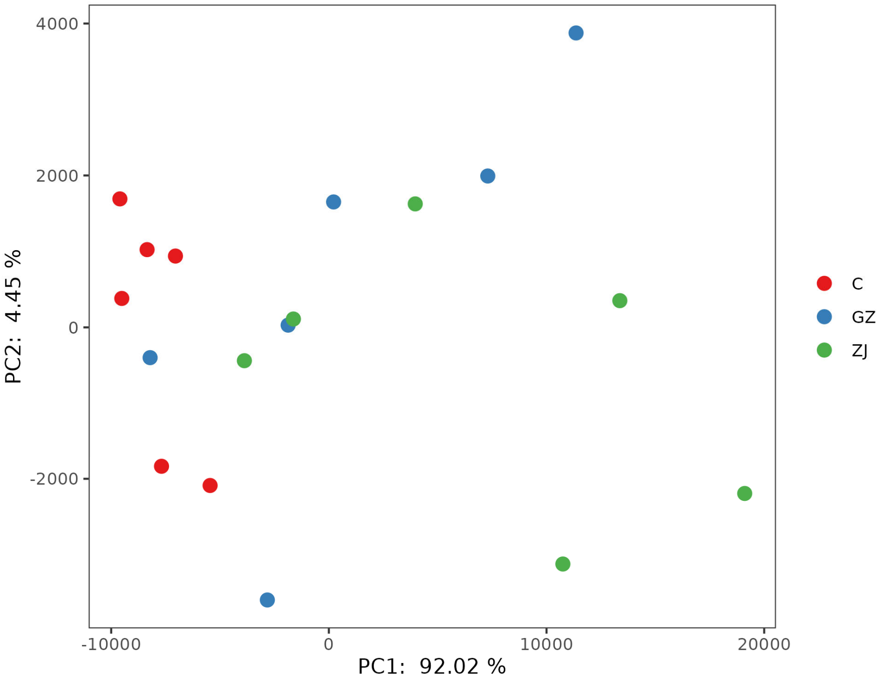

Figure S2. PCA diagram of principal component analysis at the species level.
